# Parental gambling frequency and adolescent gambling: A cross-sectional path model involving adolescents and parents

**DOI:** 10.1371/journal.pone.0280996

**Published:** 2023-02-13

**Authors:** Maria Anna Donati, Carola Beccari, Francesco Sanson, Giuseppe Iraci Sareri, Caterina Primi

**Affiliations:** 1 Department of Neuroscience, Psychology, Drug, and Child’s Health, Section of Psychology, University of Florence, Florence, Italy; 2 Gruppo Incontro, Pistoia and CEART (Coordinamento Enti Ausiliari Regione Toscana), Pistoia, Italy; Lorestan University, ISLAMIC REPUBLIC OF IRAN

## Abstract

**Introduction:**

Nowadays, most of the research studies in the field of adolescent gambling are focused on individual factors related to problem gambling. The aim of this study was to test an integrated model to explain adolescent problem gambling involving both individual (i.e., correct gambling knowledge, superstitious thinking, and gambling-related cognitive distortions) and environmental factors (i.e., parental gambling frequency). In detail, the aim was to better understand the role of parental gambling behavior on adolescents’ gambling frequency and problem gambling severity, to draw indications for prevention.

**Methods:**

Participants were 680 parents (36% men; *M*age = 48.32, *SD* = 6.14 years) of 680 high school students (51% boys; *M*age = 15.51, *SD* = .55 years) attending the second year of different high schools in Tuscany (Italy). Data collection occurred within the school prevention program *PRIZE* (Prevention of gambling risk among adolescents).

**Results:**

A path model showed was conducted to detect direct and indirect effects from individual and environmental factors to gambling problem severity. Analyses showed that correct gambling knowledge and superstitious thinking were directly related–respectively in a negative and a positive direction–both to gambling-related cognitive distortions and adolescents’ gambling frequency. Parental gambling frequency was directly associated with adolescents’ gambling frequency. Correct gambling knowledge and superstitious thinking were indirectly related to adolescents’ gambling problem severity through the mediational role of gambling-related cognitive distortions and gambling frequency. Parental gambling frequency was indirectly linked to gambling problem severity by passing through adolescents’ gambling frequency.

**Conclusions:**

The current cross-sectional results confirm that parental gambling behavior has an important role for adolescents’ gambling behavior and severity. Thus, results highlight the need for innovative prevention programs which involve adolescents’ adult reference figures and integrate the individual risk and protective factors with the environmental ones.

## 1. Introduction

Given that the current generation of youth is growing up in an era where gambling opportunities are widespread [1], and where the development of technology has generated new forms of gambling via Internet [2], nowadays gambling is part of the life experiences of most adolescents [3, 4]. Although gambling can be considered as a harmless pastime, when it becomes persistent, recurrent, and maladaptive such as to compromise personal, family or work activities, it is defined as *Gambling Disorder* (GD) [5].

Although gambling is prohibiting for youth aged less than 18 years, gamblers among adolescents across the world have been found to range from 35.7 to 74.4%, with problem gamblers ranging from 0.2 to 12.3% [3]. Some studies have also shown that adolescents’ problem gambling is higher than adults’ [e.g., 4, 6]. Problem gambling has significant psychological and social consequences on youth, such as risky and antisocial behaviors, and dysfunctional coping styles [e.g., 7, 8]. Adolescents with problem gambling present deficits in concentration and school performance, depressive symptoms, and lower self-esteem [e.g., 9, 10]. The early onset of gambling problems is also related to an increased severity of GD symptoms, more severe psychiatric symptoms, and higher rates of substance abuse in adulthood [11, 12]. Thus, the spread of gambling among adolescents and the risk for GD at this early age is a phenomenon to beware of, considering its negative consequences on the individual, the family and the society.

It has been attested that adolescent problem gambling is a multidimensional phenomenon [13, 14], which can be explained by taking into account the interwoven relationships between individuals and their environment. In detail, adolescents’ risk factors for problem gambling may be classified into two macro-areas, which include individual characteristics and environmental factors. Among the individual features, there can be mentioned low gambling related knowledge [e.g., 15, 16], high expectation of economic gain [e.g., 17, 18], superstitious thinking [e.g., 13, 19], and gambling related cognitions [20, 21]. Among environmental factors, perceived availability of gambling activities [e.g., 22], the presence of gamblers among the family [e.g., 23, 24] or in the peer group [e.g., 25, 26] can be noticed.

Over the years, much has been said about individual risk factors for adolescents, but less is known about environmental risk factors, especially concerning parental influences. In the health behavior literature, it is well documented the link between family members’ role modeling behaviors and adolescent behaviors. Studies show that parents’ attitudes towards and engagement in risky behaviors (e.g., cigarette use, alcohol use) represent risk factors for adolescents’ involvement in those behaviors [27]. Similar findings have emerged for gambling: Problem gamblers’ offspring are at increased risk for the development of gambling problems, in contrast to their peers [28, 29]. Additionally, parental gambling involvement has been associated with increased gambling frequency and gambling-related problems among adolescents [13, 30]. Parents often introduce and share gambling activities with their offspring, for example by buying scratch cards or betting with them on sport events [30, 31], thus reflecting an implicit approval, and conveying a message that gambling is a socially acceptable activity.

Despite evidence of the role of parental attitude and behaviors on adolescents’ problem gambling [e.g., 13, 30], to date few studies have investigated the role of parental gambling frequency together with a set of individual risk and protective factors. For instance, Donati and colleagues [13] found that parental gambling behavior, intended as the presence of that behavior in at least one parent, was a specific risk factor for gambling problems in adolescents, over and beyond other individual factors such as susceptibility to gambler’s fallacy, superstitious thinking, and economic positive perception of gambling. However, the frequency of gambling, rather than the mere presence of the behavior, may be a better indicator as it is the most widely used variable as a dimensional measure of gambling involvement [32]. Moreover, most studies that investigated parental gambling behavior in association with adolescents’ gambling involvement, have assessed parental gambling behavior through only the child’s perception [e.g., 13, 30], without a parental self-evaluation.

Following these premises, this cross-sectional study was aimed at investigating the role of parental gambling behavior, intended as the frequency of gambling in the last year, and measured with a self-report scale compiled by parents themselves. In particular, we asked responses from at least one parent, either the father or mother, in line with some past studies involving parents and their offspring [e.g.,^33^, ^34^]. We aimed at analyzing the relationships between some individual factors and parental gambling frequency with adolescent gambling problem severity, by hypothesizing an integrated path model. In line with Donati and colleagues’ [13] study, we employed an integrated model of protective and risky individual factors, both considering cognitive and affective dimensions, and specifically gambling-related knowledge, superstitious thinking, and gambling-related distortions. Indeed, it has been shown that knowledge about gambling is negatively related to gambling behavior, i.e., adolescents more aware of the gambling nature and harms are less involved in gambling [e.g., 15]. Instead, adolescents who are more prone to superstitious thinking are more at-risk of developing gambling problems [e.g., 19], as those who are susceptible to erroneous beliefs about the independence of random gambling events and tend to overestimate their chances of winning [e.g., 20, 35]. More in detail, we hypothesized and tested a model that integrated all the above cited research lines in order to understand a possible mechanism linking some individual risk and protective factors and parental gambling frequency to problem gambling in youth. We predicted a model in which gambling-related knowledge, superstitious thinking, and parental gambling frequency were the independent variables, and adolescent problem gambling was the dependent variable. The intermediary role was respectively exercised by gambling-related cognitive distortions—the most proximal mediator—and adolescent gambling frequency, that was thought to be the most distal mediator, in the relationship between the individual independent variables and problem gambling. Adolescent gambling frequency was the mediator between parental gambling frequency and adolescent problem gambling. The proposed model was supported by previous studies indicating that: i) gambling-related knowledge was a protective factor against the development of cognitive distortions about gambling [15, 36] and gambling behavior in youth [37]; ii) superstitious thinking was a risk factor toward the development of cognitive distortions about gambling [20] and gambling behavior in youth [38, 39]; ii) cognitive distortions related to gambling predict the frequency of gambling [13]; iii) gambling frequency can be considered as a measure of gambling involvement and a proxy measure of gambling severity [32, 40]; iii) parental gambling involvement is associated with increased prevalence of gambling and gambling-related problems among adolescents [13, 30].

We hypothesized indirect effects from the independent variables to adolescent problem gambling. Specifically, we predicted negative indirect effects from gambling-related knowledge on problem gambling, and positive indirect effects from superstitious thinking on problem gambling. Both the indirect effects were supposed to act through cognitive distortions related to gambling and adolescent gambling frequency. Indirect positive effects on GD symptoms were also expected as a result of the mediation exercised by adolescent gambling frequency between parental gambling frequency and adolescent problem gambling.

We controlled for possible relationships between gambling behavior and adolescents’ gender and age, given the associations between being boy and gambling frequently and in a problematic way [41, 42], and between age and gambling in adolescence, with the risk of becoming problem gamblers that increases once teenagers grow up and engage in a wider array of gambling activities [43]. Additionally, we took into account parents’ gender, given that men have been found to gamble more frequently than women [44].

As a preliminarily step, we aimed at describing the characteristics of gambling behavior among adolescents and their parents, investigating gambling frequency, the most engaged gambling activities, people they gamble with, and age of first gambling involvement. A more deeply understanding of parental gambling habits as well as the relationships with their offspring gambling can be useful to develop more integrated and ecological preventive programs, which take into account also protective and risk factors at the environmental level. Indeed, to date, gambling prevention programs in adolescents are mainly focused on modifying individual factors inside the school setting [see 45, 46, for reviews]. Rather, it has been recently suggested the importance of acting also on the social and ecological areas in gambling prevention [47], and, in general, parent-centered prevention for risky behaviors in adolescence is desired [e.g., 48, 49]. Precisely in this perspective of prevention, the study target were adolescents attending the second year of high school in Italy, i.e., youth aged about 15 years old. Fourteen-fifteen years has been identified as an adolescent age in which there is a high risk for gambling in the past month [49]. Moreover, although in Italy gambling is allowed solely to adolescents aged higher than 18 years, any age differences in gambling habits have been found between minor and of-age adolescents [50], indicating that even the youngest adolescents are involved in gambling.

## 2. Methods

### 2.1. Participants

Participants were 680 parents (36% men; *M*age = 48.32 years, *SD* = 6.14, range = 33.00–68.00 years) of 680 adolescents (51% boys; *M*age = 15.51 years, *SD* = .55, range = 14.00–18.00 years) attending the second year of different high schools in Tuscany (Italy). Fifty percent of students attended a lyceum, 35% a technical school, 7% a vocational school, and 2% a professional training center. In detail, 36% of the schools were located in the center of Tuscany, 35% of the schools were in the North-West of the Region, and 29% of the schools were placed in the South-East of Tuscany. For each Area of the Tuscany (North, Center, and South-East), an email presenting the prevention project was sent to all the public high schools. Attached to the email, there was a sheet to declare the participation to the project. Once the schools sent the sheet subscribed by the headmaster, they were contacted by the organizational staff of the research project and invited to specify the school classes they allowed to participate in the project. The participation was voluntary. Data collection occurred within the school prevention program *PRIZE* (Prevention of gambling risk among adolescents), during the school year 2019–2020. Seventeen percent of students reported having immigrant origins.

### 2.2. Procedure

Parents completed a paper protocol at home, which was delivered by their offspring and returned sealed to the school staff. The protocol was administered by the students to their parents in an interview-like manner, i.e., the adolescents themselves read the questions and addressed them to their parents, reporting the answers given on the protocol. Adolescents completed the survey within the classrooms and during school hours. With the aim of being able to subsequently match parents’ protocols to those of their child, the students were assigned a numerical code that had to be reported on their own protocol and on the parent’s one. Data were collected from December 2019 to February 2020.

### 2.3. Measures

#### 2.3.1. Parents

To investigate parental gambling behavior, parents were asked to indicate how often (*never*, *sometimes in the year*, *sometimes in the month*, *sometimes in the week*, *daily*) they participated in ten gambling activities in the last 12 months (card games, bets on games of personal skill, bets on sports games, bets on horse races, bingo, slot machines, scratch-cards, lotteries, online games, and private bets with friends). To obtain a score indicative of parental gambling frequency, following previous studies [e.g., 51], we attributed 0 to *never*, 1 to *sometimes in the year*, 2 to *sometimes in the month*, 3 to *sometimes in the week*, and 4 to *daily*. By summing responses to these ten items, a total score indicative of *past-year gambling frequency* can be obtained, theoretically ranging from 0 (i.e., the participant responded *never* for all the ten gambling activities) to 40 (i.e., the participant responded *daily* for all the ten gambling activities).

To analyze parental gambling versatility, i.e., the array of different gambling activities engaged in by the parents, following previous studies [e.g., 51], for each gambling activity, response options *sometimes in the year*, *sometimes in the month*, *sometimes in the week*, and *“daily”* were collapsed and scored 1, while the response option *never* was scored 0. Subsequently, the scored responses were added in order to obtain a *versatility score* theoretically ranging from 0 = any gambling participation to, to 10 = participation in all the ten listed gambling activities.

Moreover, parents were asked to report with whom they gamble (alone, with friends, with spouse, with family members), and the age of their first gambling involvement. The above-described questions were the same as administered to the offspring in Section I of the *Gambling Behavior Scale for Adolescents* (GBS-A) [52], in order to equally investigate gambling behavior in the two groups.

#### 2.3.2. Adolescents

The *Gambling Related Knowledge Scale—For Adolescents* (GRKS-A) [15] is a short self-report scale to assess adolescents’ individual knowledge about gambling, relative to its nature, functioning, and risks. It is composed by 8 Likert-type items rating on a 4-point scale, ranging from 1 (*totally disagree*), to 4 (*totally agree*). An example of item is “*In gambling*, *small winnings stimulate people to gambling again*”. The scale has good psychometric properties in adolescents. In this sample, Cronbach’s alpha was .70.

The *Superstitious Thinking Scale* (STS) [53, Italian version: 54] is a short self-report scale to assess superstitious thinking, i.e., beliefs based on perceiving biased casual relationships between unrelated events. The scale contains 8 Likert-type items, rating on a 5-point scale, ranging from 1 (*totally false*) to 5 (*totally true*). An example of item is: “*It’s bad luck if a black cat crosses your street*”. The scale has good psychometric properties [55, 56]. In this sample, Cronbach’s alpha was .82.

The *Gambling Related Cognition Scale- Revised for Adolescents* (GRCS-RA) [57, Italian version: 58] is a self-report scale to assess gambling-related cognitions in young people, which contains 14 Likert-type item rating on a 5-point scale, ranging from 1 (*strongly disagree*) to 5 (*strongly agree*). Three specific gambling-related biases, according to Toneatto’s model [59], are measured by the following subscales: *Illusion of Control* (4 items), *Predictive Control* (6 items), and *Interpretative Bias* (4 items). Respectively, an example of item is: “*In gambling praying helps you win*”, “*In gambling*, *losses are necessarily followed by a series of wins*”, “*In gambling*, *if you continue to play because if you win is thanks to personal skills and abilities*”. The scale has good psychometric properties in adolescents [60, 61]. In this sample, Cronbach’s alpha for the overall scale was .88. Cronbach’s alpha for the subscales were the following: .73 for *Illusion of Control*, .77 for *Predictive Control*, and .68 for *Interpretative Bias*.

The GBS-A [52] is a self-report scale to evaluate gambling habits and GD symptoms in adolescents. It is made up of two sections. Section I consists of unscored items investigating gambling behavior, among which gambling frequency in the last 12 months. Specifically, there are items assessing the frequency (*never*, *sometimes in the year*, *sometimes in the month*, *sometimes in the week*, *daily*) of participation during the last year in ten gambling activities (card games, bets on games of personal skill, bets on sports games, bets on horse races, bingo, slot machines, scratch-cards, lotteries, online games, and private bets with friends). Adopting the same scoring system described for the parents, a total score (theoretically ranging from 0 to 40) and indicative of past-year gambling frequency, was obtained. Consistently with what described fort the parents, a versatility score (theoretically ranging from 0 to 10) was computed.

Moreover, participants were asked to report with whom they gamble (alone, with friends, with partner, with family members), and the age of first gambling involvement.

Section II was composed by nine items, each one developed in order to relieve one of the nine symptoms listed in DSM-5, provided on a 3-Likert scale ranging from 0 (*never*) to 2 (*many times*). An example of item is: “*Have you spent in gambling money intended for other purpose*?”. The advantage of the GBS-A is that items assessing GD symptoms have been developed by applying Item Response Theory (IRT), thus it can provide a measure of gambling problem severity taking into account the severity and the discrimination power of each symptom described by the items. Moreover, the total score allows for the classification of the young gamblers in “non-disordered”, “at risk”, or “disordered”. The scale has good psychometric properties in adolescents [52, 60]. In this sample, Cronbach’s alpha for Section II was .74.

### 2.4. Ethics

To obtain the approval of this research, a study protocol in accordance with the criteria of the Declaration of Helsinki was reviewed and approved by each Head Teacher and institutional review board of the different high schools. Parents were informed with a short study description and asked to provide their informant consent. Written informed consent was obtained from all participants’ parents (both of them) to allow the participation of the adolescents. Data confidentiality was ensured according to the provisions of General Data Protection Regulation (GDPR 679/2016).

### 2.5. Statistical analyses

To describe gambling behavior habits in adolescents and their parents, we conducted descriptive statistical analyses. Then, we looked at the distribution of the variables and we carried out the path analysis. To test the hypothesized relationships among the variables, we conducted a path analysis with AMOS 16 (IBM SPSS Statistics, Armonk, NY, USA) [56] using maximum likelihood estimation. The presence of mediated effects among the variables was investigated through the test of indirect effects [62]. In AMOS, the bootstrap confidence interval method to define the confidence intervals for indirect effects [63] was implemented. In mediation analysis, bootstrapping is used to generate an empirically derived representation of the sampling distribution of the indirect effect, and this empirical representation is used for the construction of a confidence interval for the indirect effect. The 90% bias-corrected confidence interval percentile method was implemented using 2000 bootstrap samples. Confidence intervals for the indirect effects, which do not contain 0, are considered indicative of significant indirect effects, thus meaning the presence of a mediated effect. Several goodness-of-fit indices were used to test the adequacy of the model: The comparative fit index (CFI) [64], the Tucker–Lewis index (TLI) [65], and the Root Mean Square Error of Approximation (RMSEA) [66]. CFI and TLI values equal to 0.90 or greater [64, 65] and RMSEA values of.08 or below [66] are considered indices of adequate fit.

## 3. Results

### 3.1. Parents’ gambling behavior

The majority of the parents (39% men; mean age = 48.14 years; *SD* = 6.19) reported having gambled at least once in the previous 12 months. Among those who gambled, 15% (*n* = 66; 58% men; mean age = 48.66 years; *SD* = 5.79) were regular gamblers, i.e., they gambled at least one activity on a weekly or daily basis [51]. On average, parents engaged in one gambling activity. The most practiced activities were scratch-cards, lotteries, and bingo, followed by bets on sports games, card games, and online games. Parents reported mostly gambling alone (and with family members. They reported to have had the first gambling experience at about 22 years (Table 1).

**Table 1:** Gambling behavior features among adolescents and their parents (n = 680).

	*Parents*		*Adolescents*	
	*% (n)*		*% (n)*	
*Gamblers*	67 (449)		72 (456)	
*Regular gamblers*	15 (66)		48 (220)	
	*M*	*SD*	*M*	*SD*
*Versatility*	1.50	1.45	2.35	2.16
	*% (n)*		*% (n)*	
*Participation for each gambling activity*				
Card Games	9 (58)		27 (172)	
Bets on games of personal skills	3 (18)		16 (103)	
Bets on sport games	10 (69)		24 (150)	
Bet on horse races	1 (7)		5 (32)	
Bingo	30 (203)		48 (308)	
Slot machines	4 (30)		7 (41)	
Scratch cards	48 (326)		49 (312)	
Lotteries	31 (209)		17 (107)	
Online gambling activities	8 (52)		14 (91)	
Private bets with friends	4 (25)		27 (171)	
	*% (n)*		*% (n)*	
*Social partners in gambling*				
With the family	58 (215)		83 (372)	
With the partner	40 (145)		12 (49)	
With friends	52 (186)		70 (306)	
Alone	61 (228)		20 (85)	
	*M*	*SD*	*M*	*SD*
*Age of the first gambling involvement*	22.05	8.45	12.14	2.60

Note. Versatility: Score theoretically ranging from 0 = any gambling participation to, to 10 = participation in all the ten listed gambling activities. It indicates the array of different gambling activities engaged in. Participation for each gambling activity refers to the proportion of the sample (parents and adolescents) that engaged in each of the ten listed gambling activities.

### 3.2. Adolescents’ gambling behavior

The majority of adolescents (55% boys; mean age = 15.52 years; *SD* = .55) reported having gambled at least once in the previous 12 months. Among the students who reported having gambled, almost the half (68% boys; mean age = 15.60 years; *SD* = .67) were regular gamblers. On average, students engaged in two gambling activities. The most practiced activities were scratch-cards, bingo, private bets with friends, card games, and bets on sports games, followed by lotteries, and bets on games of personal skill. Students reported gambling especially with family members and friends. They have gambled for the first time at about 12 years (Table 1). Concerning gambling problem severity, 85% (*n* = 388) of adolescents turned out to be *non-disordered* gamblers, 11% (*n* = 50) *at-risk* gamblers, and 4% (*n* = 18) *disordered* gamblers.

### 3.3. Path model among individual factors, parents’ gambling frequency, and adolescent gambling behavior

The total scores distribution was significantly different from the normal distribution for all the variables considered (Correct gambling knowledge: Shapiro-Wilk test = .92, *df* = 447, *p* < .001; Superstitious thinking: Shapiro-Wilk test = .98, *df* = 447, *p* < .001; Gambling-related cognitive distortions: Shapiro-Wilk test = .92, *df* = 447, *p* < .001; Parents’ gambling frequency: Shapiro-Wilk test = .86, *df* = 447, *p* < .001.

Adolescents’ gambling frequency: Shapiro-Wilk test = .83, *df* = 447, *p* < .001; Gambling problem severity: Shapiro-Wilk test = .70, *df* = 447, *p* < .001). For each variable, a log transformation was applied. The ranges of the transformed total scores were 1.15–1.72 for correct gambling knowledge, .90–1.60 for superstitious thinking, 1.15–1.82 for gambling-related cognitive distortions, 0–1.15 for parents’ gambling frequency, 0–1.45 for adolescents’ gambling frequency, and 0–1.20 for gambling problem severity.

Pearson’s correlations were computed between adolescents’ and parents’ gender and age, individual factors–correct gambling knowledge, superstitious thinking, gambling-related distortions–parents’ gambling frequency, adolescents’ gambling frequency, and gambling problem severity (Table 2). Correlations were considered as meaningful if equal or higher than .20. Adolescents’ gambling frequency was negatively related to correct gambling knowledge, and positively related to superstitious thinking, gambling-related distortions, and parents’ gambling frequency. Adolescents’ gambling problem severity resulted to be positively associated with gambling-related cognitive distortions. Moreover, adolescents’ gambling frequency was positively correlated with adolescents’ gambling problem severity. Concerning the relationships between socio-demographic characteristics and gambling behavior, we observed a positive correlation between parents’ gender and parental gambling frequency, indicating that a mother was less at risk than a father to gamble.

**Table 2:** Summary of intercorrelations, means and standard deviations of adolescents’ gender and age, parents’ gender and age, adolescents’ individual risk and protective factors, parental gambling frequency, and adolescents’ gambling behavior.

	1.	2.	3.	4.	5.	6.	7.	8.	9.	10.
1. Adolescents’ gender	-									
2. Adolescents’ age	-.00	-								
3. Parents’ gender	.13*	-.05	-							
4. Parents’ age	-.07	-.03	-.27**	-						
5. Correct gambling knowledge	.01	-.18	.03	-.03	-					
6. Superstitious thinking	.08	.01	-.01	-.02	-.03	-				
7. Gambling-related cognitive distortions	.09	.10*	.01	-.04	-.20***	.45***	-			
8. Parents’ gambling frequency	-.05	.10*	-.21***	-.03	-.04	.08	.06	-		
9. Adolescents’ gambling frequency	-.11	.15**	-.06	-.01	-.22***	.20***	.22***	.20**	-	
10. Gambling problem severity	-.09	.16***	-.14**	.02	-.13***	.18***	.21***	.15**	.44***	-
*M*	-	15.52	-	48.31	1.43	1.25	1.42	.41	.66	.27
*SD*	**-**	.56	-	6.22	.06	.16	.14	.31	.27	.30

Note. The total scores of the variables are log-transformations. Gender: 1 = boy/man, 2 = girl/woman.

*p < .05

**p < .01

***p < .001

**Fig 1:**
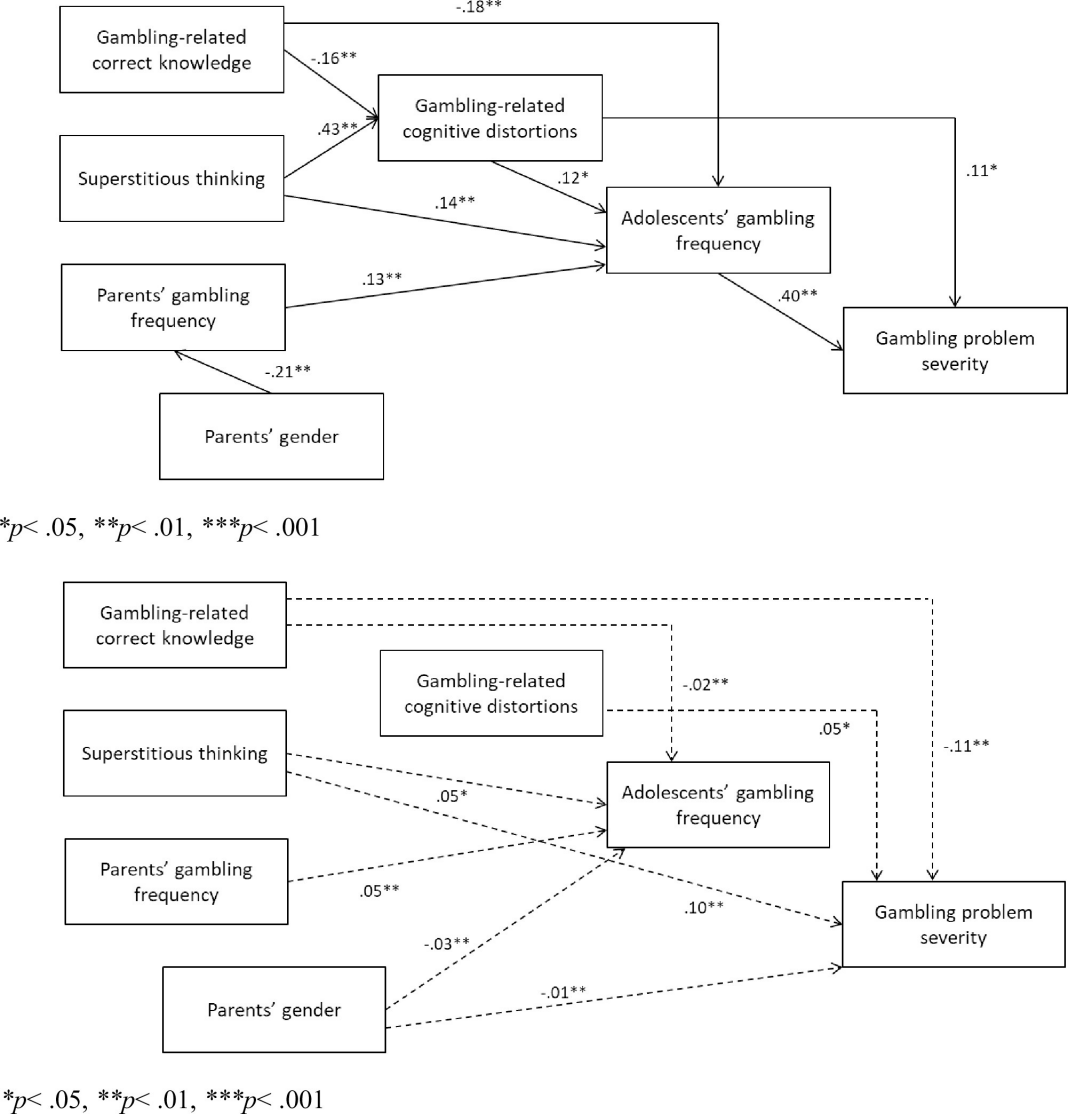
a. Path model with direct effects among the variables, and by including parents’ gender (1 = man, 2 = woman). b. Path model with indirect effects among the variables, and by including parents’ gender (1 = man, 2 = woman). *p< .05, **p< .01, ***p< .001.

Then, as correlational analyses suggested a potential meaningful relationship between parents’ gender and parental gambling frequency, we conducted the path analysis including parents’ gender in the model a covariate. Specifically, we hypothesized that it would be negatively linked to parents’ gambling frequency. Results showed that the goodness of fit indices of the proposed model were indicative of an excellent fit (CFI = .986, TLI = .927, RMSEA = .026, [90% CI 000, .059]). As hypothesized, significant and negative direct paths were found between gambling-related correct knowledge and cognitive distortions about gambling, and between gambling-related correct knowledge and adolescents’ gambling frequency. Negative direct paths emerged for superstitious thinking with respect to cognitive distortions about gambling and the frequency of gambling in adolescents. Parents’ gambling frequency exercised a significant and positive direct path on adolescents’ gambling frequency, that also received a significant and positive direct effect from gambling-related cognitive distortions. In turn, adolescents’ gambling frequency had a significant and positive effect on gambling problem severity, that was also directly linked in a positive way to cognitive distortions. Finally, the predicted direct path from parents’ gender to parental gambling frequency was significant and negative (Fig 1A).

The results also showed the following significant indirect effects: i) a negative effect from gambling-related correct knowledge to adolescents’ gambling frequency, passing by gambling-related cognitive distortions; ii) a negative effect from gambling-related correct knowledge to adolescents’ gambling problem severity, passing by gambling-related cognitive distortions and gambling frequency; iii) a positive effect from superstitious thinking to adolescents’ gambling frequency, passing by gambling-related cognitive distortions; iv) a positive effect from superstitious thinking to adolescents’ gambling frequency, passing by gambling-related cognitive distortions; v) a positive effect from parents’ gambling frequency to gambling problem severity, passing by adolescents’ gambling frequency; vi) a positive effect from gambling-related cognitive distortions to gambling problem severity, passing by gambling frequency. Moreover, parents’ gender had significant and negative indirect effects respectively on adolescents’ gambling frequency and gambling problem severity, indicating a lower likelihood for a mother, with respect to a father, that the offspring gamble frequently and in a problematic way (Fig 1B).

## 4. Discussion and conclusions

Nowadays, the widespread of gambling opportunities has allowed gambling to become part of the life experience of individuals more and more precociously. As a consequence, even if gambling is prohibiting for youth aged less than 18 years, the widespread of gambling among adolescents is high. The study aimed to more deeply investigate parental gambling habits and to better understand the role of parental gambling behavior on adolescents’ gambling frequency and GD symptoms, in order to draw indications for prevention.

Findings showed that the majority of the parents gambled at least once in the previous year, and the most practiced activities were lotteries, scratch cards, and bingo, in line with national data [67, 68] and international reports [e.g., 69, 70]. Parents mainly reported to gamble alone and with family members. Gambling alone is a behavioral habit that has to be read with caution, as gambling alone characterizes problem gambling [71, 72]. The presence of gambling behavior for the majority of the parents further supports data about the wide approval and sharing of gambling behavior in the family among youth [10, 31].

Concerning adolescents, most of them gambled at least once in the previous 12 months. On average, they engaged in two gambling activities, and the most practiced activities were scratch cards, bingo, card games, and private bets with friends and on sports games. Adolescents gambled mainly with their family members and with friends, in line with international and national studies [e.g., 3, 20]. The proportion of at-risk and disordered gambling, respectively 11% and 4%, confirm the vulnerability to gambling harms for this age group, in line with what previously reported internationally and nationally [e.g., 3, 20].

Comparing gambling features across parents and the offspring, the widespread of gambling behavior among adolescents is higher than the adults’ [e.g., 4]. Scratch-cards resulted to be the most widely engaged activity in both the groups, confirming what found by the last Italian National Research Council’s report on gambling behavior in the Italian population [73]. Although there is some evidence that scratch-cards are less risky than other types of gambling activities [74], associations between the frequency of scratch-card gambling and problem gambling severity have been demonstrated [75], and higher rates of at-risk/problem gambling have been found in those who gamble on scratch-cards compared to those who do not [76]. So, the widespread availability, popularity, and uptake of scratch-cards among youth make important to consider their potential harm.

Our path model provided further evidence that gambling behavior in youth should be read as multidimensional [e.g., 13, 14], i.e., it is important that dispositional, cognitive and social factors are considered together to provide suitable models explaining adolescents’ gambling behavior. Indeed, by integrating results from previous studies that have analyzed the relationships between some of the variables token into account in the current study [e.g., 20, 23], we showed that some cognitive, affective, and environmental factors, i.e., correct gambling knowledge, superstitious thinking exercised direct effects both on gambling-related cognitive distortions and adolescents’ gambling frequency, and parental gambling frequency have a direct effect on adolescents’ gambling frequency. Moreover, the three variables were indirectly related to adolescents’ gambling frequency: correct gambling knowledge and superstitious thinking through the mediational role of adolescents’ gambling frequency, and parental gambling frequency by passing through adolescents’ gambling frequency.

### 4.1. Practical implications

From a practical point of view, these results have practical implications for programs that aimed to prevent adolescents’ problem gambling. The proportion of gambling behavior among adolescents confirm the need to implement prevention programs that aim to reduce risk factors which increase gambling frequency which, in turns, increases the risk for problem gambling. Furthermore, results highlight the need for innovative prevention programs which involve adolescents’ adult reference figures, in line with prevention programs implemented for other risky behaviors in adolescence, such as alcohol use and substance abuse [see, for a review, 77]. In detail, prevention programs, besides aiming at reducing adolescents’ individual risk factors and at increasing protective ones, might also aim at raising parental awareness about gambling diffusion among adolescents, gambling related risk factors, and gambling psychological and social consequences on youth. Moreover, since parents often introduce to and are involved in their offspring’s gambling activities [e.g., 10, 31], prevention programs may increase parental awareness about the relevance of their behavior as a role model for adolescents. Indeed, a positive relation between perceived parental permission toward gambling and adolescents’ gambling behavior has been highlighted [e.g., 78], as well as a negative relationship between parental monitoring and problem gambling in youth [e.g., 79].

### 4.2. Limitations and future directions

The current study has a number of strengths, such as the large sample size, a standardized measure for adolescents’ gambling behavior, and the use of a self-report questionnaire to assess parental gambling behavior. However, there are several limitations. Only one parent for adolescent participated in the study. In particular, mainly mothers completed the questionnaire. This is in line with literature which indicates that the participation levels of fathers in parenting interventions are often low [e.g., 80]. Moreover, those factors related to adolescent gambling have not been investigated in parents, as well as parental gambling severity. The study involved a sample of Italian public-school students of Tuscany, thus, some limitations regarding external validity and generalizability might be related to the specificity of the sample. Additionally, we had not information regarding parental socio-economic status and education level, while these variables could represent confounding factors in the relationship between parental gambling behavior and adolescent gambling behavior. As this is a cross-sectional study, the paths found among the variables must be read as associations, without draw any cause-effect relationships. Future studies could analyze the relationship between parents’ and adolescents’ individual risk factors, and the relationship between parental gambling severity and gambling behavior in youth. Additionally, the role of parental attitude toward gambling and parental monitoring on adolescents’ gambling behavior might be investigated, especially through longitudinal research designs. Finally, it should be important to take into account also parental gambling problem severity by applying a research design in which parents are involved in responding to online questionnaires, for instance, without the intermediation of adolescents.

## Supporting information

S1 Dataset (SAV)
